# Cell phone based telemedicine - brief introduction

**DOI:** 10.1186/1479-5876-10-S2-A47

**Published:** 2012-10-17

**Authors:** Chunxue Bai

**Affiliations:** 1 Shanghai Respiratory Research Institute (SRRI), Shanghai, 200032, China; 2 Department of Pulmonary medicine, Zhongshan Hospital, Fudan University, Shanghai, 200032, China

## 

In 2008, cell phone user population hit 641 million while the mobile web user population reached 117 million in China. The development of mobile technology gives rise for greater potential to improve the public’s health. Leveraging on the development of intelligent sensors, wireless transmitters, as well as a dynamic medical network, we bring forth a new concept of cell phone-based telemedicine specially catered for those suffering from chronic respiratory diseases. Such a cost effective and convenient health care service is enabled though the utilization of wifi/GPRS (General Packet Radio Service) system and GPS (Global Positioning System) in widely used cell phones. Briefly, our cell phone based medical service is composed with the following components: (1) Terminal devices collecting information from the patient, like oxygen saturation, heart rate, lung function or other physiological parameters. The breakthrough of our system is to integrate the spirometer with a cell phone unit (ATS NEWS, 2009, VOL.35 NO.7/8). (2) Electronic transfer of such information over a distance through the internet wirelessly; (3) Terminal devices by which doctors remotely receive patients’ information and send feedback or tailored advice to the patients. (4) A medical center for coordinating, providing technical services and regulating. (5) Software enabling the interactive communication between doctors and patients.

In summary, Cell phone based Telemedicine could monitor diseases and facilitate personalized management in mobile way with following advantage: real time, quick, high efficiency; reduce hospital visits and improve outcome; cost-effective in the long-term; alert the patients to take medicine or measurement. It will also Increase working efficiency of famous doctors, could educate doctors and patients by mobile way, and could work for Clinical Trial.

